# Spatiotemporal Characteristics of the Largest HIV-1 CRF02_AG Outbreak in Spain: Evidence for Onward Transmissions

**DOI:** 10.3389/fmicb.2019.00370

**Published:** 2019-03-11

**Authors:** Evangelia-Georgia Kostaki, Andreas Flampouris, Timokratis Karamitros, Natalia Chueca, Marta Alvarez, Paz Casas, Belen Alejos, Angelos Hatzakis, Federico Garcia, Dimitrios Paraskevis, CoRIS, Santiago Moreno

**Affiliations:** ^1^Department of Hygiene, Epidemiology and Medical Statistics, Medical School, National and Kapodistrian University of Athens, Athens, Greece; ^2^Department of Zoology, University of Oxford, Oxford, United Kingdom; ^3^Public Health Laboratories, Department of Microbiology, Hellenic Pasteur Institute, Athens, Greece; ^4^Department of Clinical Microbiology, Hospital Universitario San Cecilio, Instituto de Investigación Ibs, Granada, Spain; ^5^Centro Nacional de Epidemiología, Universidad de Alcalá de Henares, Madrid, Spain

**Keywords:** HIV-1, CRF02_AG, Spain, regional dispersal, spatiotemporal characteristics

## Abstract

**Background and Aim:** The circulating recombinant form 02_AG (CRF02_AG) is the predominant clade among the human immunodeficiency virus type-1 (HIV-1) non-Bs with a prevalence of 5.97% (95% Confidence Interval-CI: 5.41–6.57%) across Spain. Our aim was to estimate the levels of regional clustering for CRF02_AG and the spatiotemporal characteristics of the largest CRF02_AG subepidemic in Spain.

**Methods:** We studied 396 CRF02_AG sequences obtained from HIV-1 diagnosed patients during 2000–2014 from 10 autonomous communities of Spain. Phylogenetic analysis was performed on the 391 CRF02_AG sequences along with all globally sampled CRF02_AG sequences (*N* = 3,302) as references. Phylodynamic and phylogeographic analysis was performed to the largest CRF02_AG monophyletic cluster by a Bayesian method in BEAST v1.8.0 and by reconstructing ancestral states using the criterion of parsimony in Mesquite v3.4, respectively.

**Results:** The HIV-1 CRF02_AG prevalence differed across Spanish autonomous communities we sampled from (*p* < 0.001). Phylogenetic analysis revealed that 52.7% of the CRF02_AG sequences formed 56 monophyletic clusters, with a range of 2–79 sequences. The CRF02_AG regional dispersal differed across Spain (*p* = 0.003), as suggested by monophyletic clustering. For the largest monophyletic cluster (subepidemic) (*N* = 79), 49.4% of the clustered sequences originated from Madrid, while most sequences (51.9%) had been obtained from men having sex with men (MSM). Molecular clock analysis suggested that the origin (t_MRCA_) of the CRF02_AG subepidemic was in 2002 (median estimate; 95% Highest Posterior Density-HPD interval: 1999–2004). Additionally, we found significant clustering within the CRF02_AG subepidemic according to the ethnic origin.

**Conclusion:** CRF02_AG has been introduced as a result of multiple introductions in Spain, following regional dispersal in several cases. We showed that CRF02_AG transmissions were mostly due to regional dispersal in Spain. The hot-spot for the largest CRF02_AG regional subepidemic in Spain was in Madrid associated with MSM transmission risk group. The existence of subepidemics suggest that several spillovers occurred from Madrid to other areas. CRF02_AG sequences from Hispanics were clustered in a separate subclade suggesting no linkage between the local and Hispanic subepidemics.

## Introduction

Human immunodeficiency virus (HIV)-pandemic has been caused by Group M which is divided into nine subtypes (A, B, C, D, F, G, H, J, and K), six sub-subtypes (A1, A2, A3, A4, F1, F2) but also an extensive list of at least 98 CRFs which result from the recombination of two or more different subtypes (Foley et al., 2016)^1^. The distribution of subtypes and CRFs differ greatly across the globe. The majority (46.6%) of the infections worldwide are caused by subtype C, followed by subtype B (12.1%) and subtype A (10.3%); the former is predominant in the Western world (Hemelaar et al., 2018). CRF02_AG is the fourth worldwide (7.7%), however, it accounts for approximately 50% of the HIV-infections in West and Central Africa (Delatorre et al., 2014). CRF01_AE (5.3%), subtype G (4.6%) and D (2.7%) are following (Hemelaar et al., 2018).

The putative origin of CRF02_AG was in Central Africa, thereafter the virus spread into Western Africa establishing a major epidemic (Mir et al., 2016). Further dissemination from Western Africa occurred to Cameron in the late seventies, to the former Soviet Union in the late nineties and later to Bulgaria and Germany (Mir et al., 2016). CRF02_AG remains one of the most prevalent CRFs in Europe among individuals from highly endemic countries but also in non-migrant populations (Abecasis et al., 2013; Beloukas et al., 2016).

A previous study on 6,633 sequences sampled across Spain, revealed that subtype B was the most prevalent, but the non-B clades were dominated by CRFs. Specifically, it was found that CRF02_AG had a prevalence of 5.97% (95% CI: 5.41–6.57%) and was the most prevalent among the non-B clades (Flampouris et al., 2014). It is estimated that 130,000–160,000 people are living with HIV in Spain (Morán Arribas et al., 2018) while there is a high burden of late diagnosis. Current HIV epidemic in Spain is dominated by infections among MSM (del Amo and Iniesta, 2018).

The aim of the current study was to estimate the levels of regional clustering for CRF02_AG in Spain, to assess parameters associated with its regional dispersal across Spain, but also to estimate the spatiotemporal characteristics of the largest CRF02_AG subepidemic in Spain. We also investigated whether regional dispersal is due to migration or due to onward transmissions among non-migrants.

## Materials and Methods

### Study Population

We studied 396 HIV-1 CRF02_AG sequences available in the protease and partial reverse transcriptase regions of the virus genome (*pol gene*). Sequences were obtained from HIV-1 diagnosed patients during 2000–2014 from 10 autonomous communities of Spain. Study population characteristics are shown in Table 1. Specifically, patients’ samples were merged from two datasets: (a) a representative cohort of HIV-infected patients included in the Research Network on HIV/AIDS (CoRIS) (2004–2013) and (b) Eastern Andalusia Resistance Cohort (2000–2014). A detailed description of the CoRIS cohort has been published previously (Caro-Murillo et al., 2007; Yebra et al., 2012). For this study, fully anonymized nucleotide sequences were retrospectively analyzed thus no written informed consent is required. This research project has been approved by the HU san Cecilio’s Ethics Committee.

**Table 1:** Characteristics of the study population: (i) total population (*N* = 396), and (ii) a subset of the total population (*N* = 391 of 396) after the exclusion of identical sequences.

Characteristic	Total population	Subset of the total population
Median age [years (IQR^1^)]	35.6 (29.3–43.6)^2^	36.2 (29.4–43.6)^3^
**Gender (N, %)**		
Male	233 (58.8)	230 (58.8)
Female	163 (41.2)	161 (41.2)
**Transmission risk group (N, %)**		
Heterosexuals	158 (39.9)	156 (39.9)
MSM^4^	62 (15.7)	60 (15.4)
PWID^5^	1 (0.2)	1 (0.2)
Other/unknown	175 (44.2)	174 (44.5)
**Spanish autonomous community of sampling (N, %)**		
Andalusia	240 (60.6)	239 (61.3)
Madrid	76 (19.2)	75 (19.2)
Valencia	22 (5.6)	19 (4.9)
Catalonia	18 (4.6)	18 (4.6)
La Rioja	11 (2.8)	11 (2.8)
Navarre	11 (2.8)	11 (2.8)
Basque Country	11 (2.8)	11 (2.8)
Balearic Islands	3 (0.7)	3 (0.7)
Galicia	3 (0.7)	3 (0.7)
Canary Islands	1 (0.2)	1 (0.2)
**Total (N, %)**	**396 (100)**	**391 (100)**


*^1^IQR, interquartile range; ^2^N = 313 (of 396 individuals); ^3^N = 309 (of 391 individuals); ^4^MSM, men having sex with men; ^5^PWID, people who inject drugs.*

### DNA Sequence Alignment and Phylogenetic Analysis

The patterns of CRF02_AG dispersal in Spain were investigated by means of phylogenetic analysis. Analysis was performed in 391 out of 396 (98.7%) CRF02_AG sequences along with all globally sampled CRF02_AG sequences available on the public HIV-1 sequence database^2^ (*N* = 3,302) as references. A small number of identical sequences (5 of 396, 1.3%) was excluded from analysis to avoid inclusion of duplicates. MEGA v5.2 (Hall, 2013) was used to align sequences using the MUSCLE algorithm and alignments were manually edited according to the encoded reading frame (only codons and no single or double combinations of nucleotides were excluded from the alignment). Codons associated with resistance (IAS mutation list 2017) were excluded to avoid potentially bias on clustering due to convergent evolution at resistance sites (Lewis et al., 2008). The final alignment was consisted of 738 nucleotides. FigTree v1.4 was used for tree visualization and annotation^3^.

The phylogenetic trees were estimated from the underlying nucleotide sequences. Initially, Maximum likelihood phylogeny reconstruction with bootstrap evaluation was conducted in RAxML v8.0.20 (Stamatakis, 2014) using the general time-reversible (GTR) substitution model and gamma (Γ) distribution. Subsequently, further analysis was performed on the clusters that initially received bootstrap value lower than 75%, by using a well-justified method based on Bayesian analysis (Kouyos et al., 2010; Paraskevis et al., 2019). In detail, we used the Simple Consensus Maker algorithm^4^ to make the consensus sequence of each cluster with bootstrap value lower than the threshold. The 100 most closely related sequences were found by ordering the consensus sequence with all the CRF02_AG available sequences (391 sequences from our study population and 3,302 reference sequences) according to their similarity, in Mafft v7.4 (Katoh et al., 2017). Sequences found within each cluster were then analyzed phylogenetically along with the 100 most closely related sequences to them, using the Bayesian method with the GTR substitution model with Γ distributed rate, as implemented in MrBayes v3.2.2 (Ronquist and Huelsenbeck, 2003). The MCMC ran for 10 × 10^5^ generations (burn-in: 10%), with 4 chains per run, and with MCMC sampling every 1,000 steps. Each MCMC run checked for convergence using Tracer version v1.5 (Drummond et al., 2012). Thereafter, phylogenetic clusters were defined as monophyletic using two different criteria: (i) clusters with bootstrap values greater than 75%, for phylogenetic trees estimated by the Maximum likelihood method, or a posterior probability greater than 0.82^5^, for phylogenetic trees estimated by the Bayesian method (phylogenetic confidence criterion), and (ii) clusters consisting of at least two sequences sampled from Spain at a proportion greater than 70% compared to total number of sequences within the cluster (geographic criterion). Only phylogenetic clusters fulfilling both criteria were considered as monophyletic.

### Phylodynamic Analysis

We further conducted phylodynamic analysis to estimate the spatiotemporal characteristics of the largest CRF02_AG subepidemic (monophyletic cluster consisted of 79 sequences). Analysis was performed only on sequences with available information about their date of sampling (72 of 79, 91.1%). We performed the analysis by a Bayesian method using the HKY nucleotide substitution model, a γ distributed rate of heterogeneity among sites, an uncorrelated log normal relaxed clock of molecular clock model with TipDates and the birth-death basic reproductive number (R_e_) models as implemented in BEAST v1.8.0 (Drummond et al., 2012). Non-informative priors were used for the MCMC runs. MCMC analysis was run for 30 × 10^6^ generations, sampled every 3.000 steps (burn-in: 10%). The MCMC convergence was checked using Tracer v1.5 (Drummond et al., 2012), by estimating the effective sample sizes (ESS > 200). The temporal signal in our data was tested by TempEst (Rambaut et al., 2016).

### Phylogeographic Analysis

Phylogeographic analysis was inferred by character reconstruction using the criterion of parsimony on the dated phylogeny using Mesquite v3.4^6^.

### Statistical Analysis

Demographic data are summarized using median and interquartile ranges for continuous variables, and absolute and relative frequencies for categorical variables. Statistical analysis for simple comparisons of the relevant distributions across different levels of other categorical variables was carried out using Pearson’s chi-squared statistical test. A multivariate logistic regression model was fit to a subset of the original data (*N* = 391), consisting of 304 complete observations. Presence in monophyletic groups was the binary outcome variable, while age, gender, transmission risk group and Spanish autonomous community of sampling were chosen as possible explanatory variables. Statistical analysis was performed on STATA 12-StataCorp LP.

## Results

CRF02_AG prevalence was significantly different across the Spanish autonomous communities we sampled from (*p* < 0.001). La Rioja (*N* = 11, 16.7%, 95% CI: 8.6–27.9%), Navarre (*N* = 11, 13.6%, 95% CI: 7–23%), Valencia (*N* = 22, 7.3%, 95% CI: 5.3–12.4%), and Andalusia (*N* = 240, 7%, 95% CI: 6.2–8.0%) were the autonomous communities where CRF02_AG was most prevalent.

**FIGURE 1:**
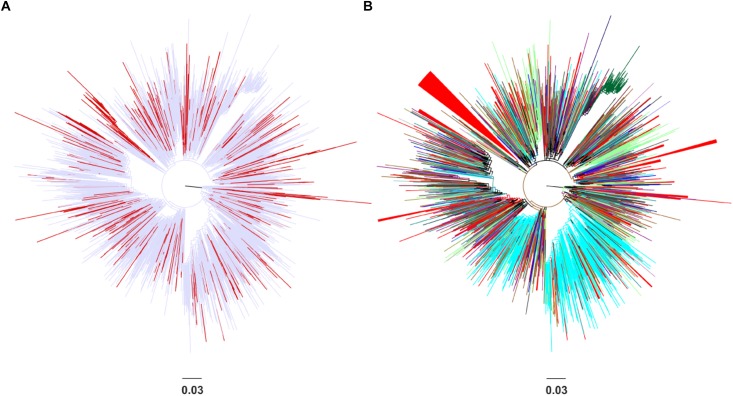
Unrooted phylogenetic trees estimated by RAxML v8.0.20 of HIV-1 CRF02_AG sequences sampled from Spain and a global reference dataset. **(A)** Sequences from Spain are marked in red in contrast with sequences from other areas marked in light blue. **(B)** Sequences from Spain are marked in red in contrast with sequences from other countries (Cameroon, Senegal, Niger, Ghana, Mali, the United States, Burkina Faso, Togo, Italy, Russia, Benin, Gabon, Germany, Belgium, the United Kingdom, Japan, France, Mauritania, Portugal, Sweden, Equatorial Guinea, the Czech Republic, Denmark, Cyprus, Australia, and Canada) and geographical areas (Western Europe, Sub-Saharan Africa, Central Asia, East Asia, Central and Eastern Europe, Southeast Asia, and Latin America) marked in different colors. Monophyletic clusters according to geographic region of sampling are indicated as triangles.

Phylogenetic analysis revealed that the CRF02_AG sequences sampled from Spain clustered at different points in the Maximum-likelihood tree (Figure 1A). No specific geographic area was identified as the origin of CRF02_AG in Spain, since the Spanish monophyletic clusters appeared as nested within sequences from different countries (Figure 1B). The high-levels of dispersal of the CRF02_AG strains (red branches) across the tree (Figure 1A) or the existence of a large number of monophyletic clusters (Figure 1B) indicate that the introduction of CRF02_AG into Spain has been occurred from multiple sources. Specifically, we found that 52.7% (206 of 391) of our study CRF02_AG sequences formed 56 monophyletic clusters, with a range of 2–79 sequences (Figure 1B). The median number of sequences per monophyletic cluster was 3 (Interquartile range, IQR: 2–5). Phylogenetic analysis revealed the presence of highly supported phylogenetic clusters consisted of sequences from Spain at proportions >70% (monophyletic clusters). HIV-1 sequences found within the monophyletic clusters suggest a common route of infection. The fact that the majority of sequences have been retrieved from PLHIV residing in Spain implies that monophyletic clusters correspond to transmissions occurred locally (regional transmissions).

**FIGURE 2:**
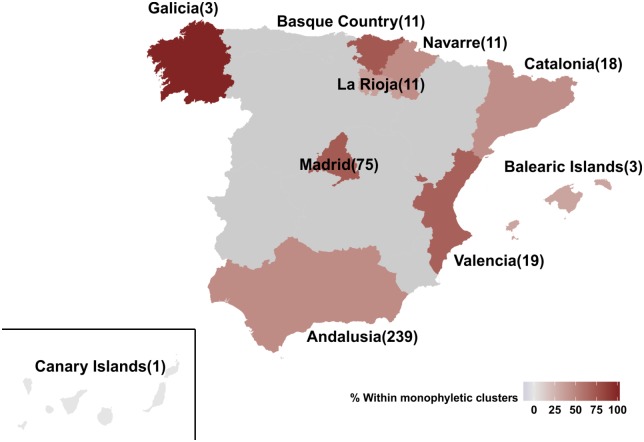
Map of Spain representing the 10 autonomous communities from which the 391 HIV-1 CRF02_AG sequences of our study population were sampled from. Autonomous communities are colored according to the percentage of monophyletic clustering. Numbers in brackets describe the available sequences per autonomous community.

The percentage of regional dispersal differed across Spain, as suggested by the monophyletic clustering (*p* = 0.003) (Figure 2). Specifically, the highest proportion of regional dispersal was found in Basque Country where 72.7% (8 of 11) of sequences belonged to monophyletic clusters, followed by sequences from Madrid (54 of 75, 72%) and Valencia (13 of 19, 68.4%). Sequences from Andalusia (110 of 239, 46%), Navarre (5 of 11, 45.5%), Catalonia (8 of 18, 44.4%), and La Rioja (4 of 11, 36.4%) showed the lowest monophyly levels. For Galicia (*N* = 3), Canary Islands (*N* = 1), and Balearic Islands (*N* = 3) less than 10 sequences were available. The current study was performed using data from the representative cohort CoRIS (Caro-Murillo et al., 2007; Yebra et al., 2012; Vourli et al., 2019) and the Eastern Andalusia Resistance Cohort. The study population included sequences sampled during 2000–2014 from 10 autonomous communities of Spain, suggesting high geographic coverage. The proportion of sequences within each cluster was comparable across different communities, expect for Andalusia which was overrepresented. Despite its over-representativeness, Andalusia was found among the communities with the lowest levels of monophyly.

Using a multivariate logistic regression model, we found that the MSM transmission risk group was positively associated with the regional clustering (*p* < 0.001), having adjusted for the rest of the incorporated variables (Table 2). This association was mostly due to the large monophyletic cluster in which the majority of PLHIV were MSM.

**Table 2:** Multivariate logistic regression estimates using the presence in monophyletic groups as the binary outcome variable.

Explanatory variable	Odds ratio	95% Conf. interval	*P*-value
Age	1.01	(0.99, 1.03)	0.506
**Gender (^∗^Male)**			
Female	1.09	(0.65, 1.84)	0.732
**Transmission risk group (^∗^Heterosexuals)**			
MSM^1^	6.66	(2.57, 17.23)	<0.001
PWID^2^	1	–	–
Other/unknown	0.63	(0.34–1.18)	0.153
**Spanish autonomous community of sampling (^∗^Andalusia)**			
Madrid	0.88	(0.41, 1.91)	0.749
Valencia	1.04	(0.33, 3.22)	0.948
Catalonia	0.45	(0.15, 1.34)	0.152
La Rioja	0.48	(0.12, 1.90)	0.299
Navarre	0.47	(0.12, 1.82)	0.277
Basque Country	1.19	(0.26, 5.29)	0.823
Balearic Islands	0.33	(0.03, 3.93)	0.381
Galicia	1	–	–
Canary Islands	1	–	–


*The model was fit to a subset of the original data (N = 391), consisting of 304 complete observations. ^∗^Reference category: ^1^MSM, men having sex with men; ^2^PWID, people who inject drugs.*

The largest CRF02_AG monophyletic cluster (subepidemic) consisted of 79 sequences (Figure 1B). We found that 49.4% (39 of 79) of the clustered sequences originated from Madrid and most sequences (41 of 79, 51.9%) had been obtained from MSM. Phylogenetic analysis also revealed the existence of two nested clusters from Japan (*N* = 7, 8.9%) and Sweden (*N* = 3, 3.8%). The nested clusters consisted of reference sequences that were available in the HIV-1 sequence database.

**FIGURE 3:**
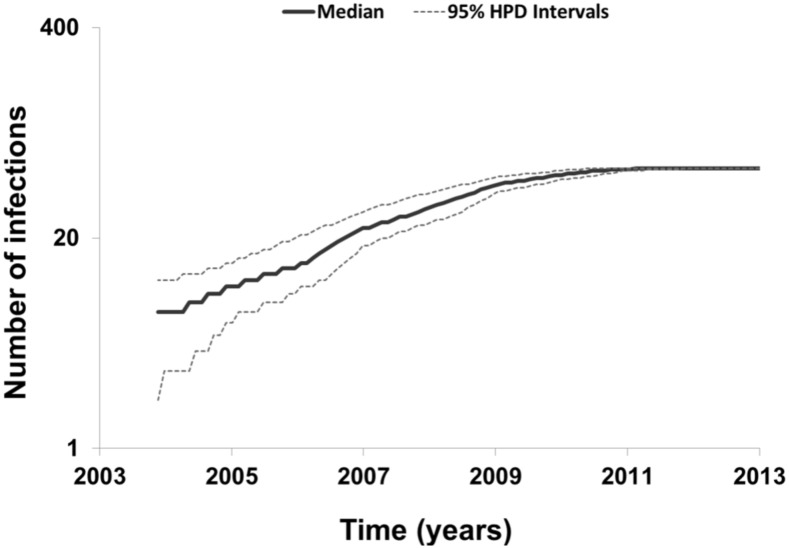
Bayesian skyline plot estimated by BEAST v1.8.0 using birth-death models for the largest HIV-1 CRF02_AG subepidemic in Spain, showing the cumulative number of lineages (infections) in a logarithmic scale over time (median and 95% Highest Posterior Density-HPD intervals estimates).

To describe the temporal patterns of the largest CRF02_AG regional epidemic in Spain, we carried out phylodynamic analysis. There was evidence for temporal signal in the sequences found within the largest CRF02_AG monophyletic cluster, as tested by the TempEst program (*R* = 0.721). Moreover, the temporal structure in this data was assessed by estimating the ESS for all parameters of the MCMC analysis. In the MCMC analysis the ESS for all parameters were >>200. As explained in detail in the methods section, phylodynamic analysis was performed only on sequences with available information about their date of sampling (*N* = 72). For 57 out of 72 (79.2%) sequences found within the largest CRF02_AG monophyletic cluster, the sampling area was in Spain (Table 3). Molecular clock analysis suggested that the time to most recent common ancestor (t_MRCA_) of the CRF02_AG subepidemic was in 2002 (median estimate; 95% Highest Posterior Density-HPD interval: 1999–2004). The t_MRCA_ should be considered as the approximate time of infection of the potential founder of the CRF02_AG subepidemic in Spain sampled in our data. The Bayesian skyline plot showed that the CRF02_AG subepidemic growth occurred during a period of 8 years and specifically between 2003 and 2011 (Figure 3).

**Table 3:** Sampling region/country for sequences with known date of sampling found within the largest CRF02_AG monophyletic cluster (subepidemic) in Spain.

Region of sampling	Country of sampling	Number of sequences per country (%)	Number of sequences per region (%)
Western Europe	Spain	57 (79.2)	65 (90.3)
	Switzerland	4 (5.6)	
	Sweden	3 (4.2)	
	Germany	1 (1.2)	
East Asia	Japan	7 (9.8)	7 (9.7)
**Total**		**72 (100)**	**72 (100)**


**FIGURE 4:**
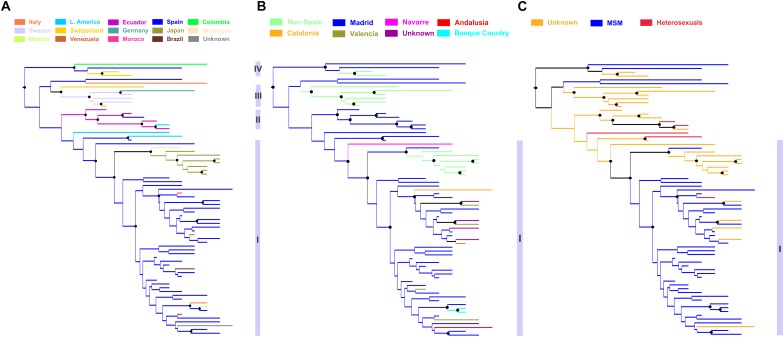
Phylogeographic topology estimated by Mesquite v3.4 for largest HIV-1 CRF02_AG subepidemic in Spain, showing the viral clades in different colors according to the: **(A)** ethnic origin of the HIV-1 diagnosed patient, **(B)** Spanish autonomous community of sampling, and **(C)** transmission risk group of the HIV-1 diagnosed patient. Branch lengths were longer within subclusters III, IV than within subclusters I and II, suggesting higher genetic diversity. Black disks indicate nodes with posterior probability greater than 0.85.

Phylogeographic analysis revealed significant clustering within the CRF02_AG subepidemic according to the ethnic origin (Figure 4A). Specifically, four distinct subclades were found. The subclade division was based on high posterior probability support (>0.85) and epidemiologic criteria (i.e., the ethnic origin of PLHIV within each cluster). The first subclade was consisted of sequences from Spain (*N* = 44) including as nested the cluster from Japan (*N* = 7) (subclade I) (Figure 4A,B). The majority of Spanish lineages were from individuals living in Madrid (*N* = 30, 68.2%) reported MSM (*N* = 37, 84.1%) as transmission risk group (Figure 4B,C). Except from Madrid, Spanish sequences were obtained across five different autonomous communities of Spain (Figure 4B). The second subclade was consisted of sequences from Hispanics from Spain (*N* = 1, 17%) and Latin America (*N* = 5, 83%) (subclade II) (Figure 4A). The third subclade consisted of sequences (*N* = 6) originating from Western Europe (Sweden, Switzerland, and Germany) (subclade III) and the fourth of sequences (*N* = 4) from Colombia, Spain, and Switzerland (subclade IV) (Figure 4A). Subclades III and IV were more genetically divergent than the two others (as suggested from the within-subcluster branch lengths) (Figure 4A). Notably, the geographic origin of the CRF02_AG clade including sequences from Spain (subclades I and II) was estimated in Madrid, from where it spread to other autonomous communities within the country as well as outside Spain (Figure 4A,B).

## Discussion

In this study we used a collection of sequences sampled from 10 Spanish autonomous communities and a combination of phylogenetic, phylogeographic and phylodynamic analyses, in order to investigate the characteristics of the HIV-1 CRF02_AG epidemic in this country. We further shed light on the spatiotemporal characteristics of the largest CRF02_AG subepidemic in Spain. Our analysis suggests that CRF02_AG has been introduced as a result of multiple introductions in Spain, following regional dispersal in several cases. However, we found considerable variation in the patterns of regional clustering in Spain with Basque area, Madrid and Valencia to be the areas with the higher proportion of regional transmissions. We estimated that the epidemic growth of the largest monophyletic cluster continued until 2011. The hub for the largest cluster was in Madrid from where cross-border and local transmissions to other autonomous communities were monitored. Notably, CRF02_AG sequences within the largest cluster in Madrid were detected as far as from Japan, comprising a local epidemic, as well as from Latin America. Although an alternative hypothesis cannot be entirely excluded, our suggestion that the Japanese local epidemic originated from Spain provides the most plausible hypothesis given the existing sampling. The long-distance dispersal of CRF02_AG is not strange since some of the sequences were obtained from Hispanic populations outside Spain, a finding that can be reconciled because of the historical links between Spain and Hispanic populations of the Americas. On the other hand, the high popularity of Spain as a travel destination could explain the outgoing transmissions to Japan. CRF02_AG regional dispersal in Spain was associated with MSM; a finding that was probably due to the pattern found for the largest cluster in Madrid.

The identification of Madrid as a hub for the largest cluster of CRF02_AG in Spain and that only small clusters were found outside Madrid, suggest that transmission networking is probably more extensive in the capital than in peripheral cities. Notably, similar findings were reported for Paris and Rome which provided as the source for the CRF02_AG transmissions across France and Italy, respectively (Giuliani et al., 2013; Chaillon et al., 2017). The identity of capital cities as hubs for within country dispersal could be explained by the fact that they provide social, economic cultural and recreational centers as well as major hubs for international traveling. These findings could be useful for public health stakeholders to focus their preventive actions in large cities in Europe.

The prevalence of CRF02_AG has been estimated to 3.0% in Western and Central Europe and North America, being the most frequently circulated among the CRFs (Hemelaar et al., 2018). Although sporadic CRF02_AG cases have been increasingly detected in Europe and North America as a result of population mobility from endemic regions, regional dispersal was also found in several areas in Europe including MSM and heterosexual populations in France, Belgium, Switzerland, and Spain (Dauwe et al., 2015), as well as people who inject drugs in Bulgaria (Alexiev et al., 2016). Specifically, only one small cluster was found in Belgium, while 28% of the CRF02_AG sequences belonged to Swiss-specific subclusters (Von Wyl et al., 2011). In France, 20.4% of the PLHIV primarily infected with CRF02_AG belonged to transmission networks based on analysis using a genetic distance cut-off (Chaillon et al., 2017). This proportion was the highest for all HIV-1 subtypes (Chaillon et al., 2017). A large monophyletic cluster of CRF02_AG was also monitored among PLHIV with primary infection in Southeastern France (Tamalet et al., 2015). Similarly, the proportion of CRF02_AG sequences within monophyletic clusters was reported: 29.8, 27.3, 25.4, and 18.2% for Germany, Norway, Austria, and Italy, respectively (Paraskevis et al., 2019). These findings suggest a common pattern of CRF02_AG dispersal in Europe, where although CRF02_AG have been associated with highly endemic areas (Abecasis et al., 2013) as their putative source, a considerable proportion of their sequences fall within monophyletic clusters. It remains unclear, however, whether onward transmissions in Europe occur among migrant or non-migrant populations.

In the current study using a state-of-the-art molecular epidemiology approach, we were able to describe the spatiotemporal characteristics of the CRF02_AG infection in Spain and to investigate the nature of onward transmissions across different ethnic groups. Notably, we found that the majority of CRF02_AG sequences within the largest cluster were retrieved from non-migrant populations, suggesting that the within the country infections are due to onward transmissions among non-migrants. To our knowledge this is one of the few studies providing evidence that local dispersal of non-B subtypes is associated with non-migrants. These findings can be important for public health and particularly for the design of targeted interventions for the populations at the higher risk.

## Centers and Investigators Involved In CoRIS

### Executive committee

Santiago Moreno, Inma Jarrín, David Dalmau, Maria Luisa Navarro, Maria Isabel González, Jose Luis Blanco, Federico Garcia, Rafael Rubio, Jose Antonio Iribarren, Félix Gutiérrez, Francesc Vidal, Juan Berenguer, Juan González.

Fieldwork, data management and analysis: Belén Alejos, Victoria Hernando, Cristina Moreno, Carlos Iniesta, Luis Miguel Garcia Sousa, Nieves Sanz Perez.

### BioBanK HIV

Hospital General Universitario Gregorio Marañón: M Ángeles Muñoz-Fernández, Isabel María García-Merino, Irene Consuegra Fernández, Coral Gómez Rico, Jorge Gallego de la Fuente, Paula Palau Concejo.

### Participating Centres

Hospital General Universitario de Alicante (Alicante):

Joaquín Portilla, Esperanza Merino, Sergio Reus, Vicente Boix, Livia Giner, Carmen Gadea, Irene Portilla, María Pampliega, Marcos Díez, Juan Carlos Rodríguez, José Sánchez-Payá.

Hospital Universitario de Canarias (San Cristobal de la Laguna): Juan Luis Gómez, Jehovana Hernández, María Remedios Alemán, María del Mar Alonso, María Inmaculada Hernández, Felicitas Díaz-Flores, Dácil García, Ricardo Pelazas, Ana López Lirola.

Hospital Universitario Central de Asturias (Oviedo): José Sanz Moreno, Alberto Arranz Caso, Cristina Hernández Gutiérrez, María Novella Mena.

Hospital Universitario 12 de Octubre (Madrid): Rafael Rubio, Federico Pulido, Otilia Bisbal, Asunción Hernando, Lourdes Domínguez, David Rial Crestelo, Laura Bermejo, Mireia Santacreu.

Hospital Universitario de Donostia (Donostia-San Sebastián): José Antonio Iribarren, Julio Arrizabalaga, María José Aramburu, Xabier Camino, Francisco Rodríguez-Arrondo, Miguel Ángel von Wichmann, Lidia Pascual Tomé, Miguel Ángel Goenaga, Ma Jesús Bustinduy, Harkaitz Azkune, Maialen Ibarguren, Aitziber Lizardi, Xabier Kortajarena.

Hospital General Universitario De Elche (Elche): Félix Gutiérrez, Mar Masiá, Sergio Padilla, Andrés Navarro, Fernando Montolio, Catalina Robledano, Joan Gregori Colomé, Araceli Adsuar, Rafael Pascual, Marta Fernández, Elena García, José Alberto García, Xavier Barber.

Hospital General Universitario Gregorio Marañón (Madrid): Juan Berenguer, Juan Carlos López Bernaldo de Quirós, Isabel Gutiérrez, Margarita Ramírez, Belén Padilla, Paloma Gijón, Teresa Aldamiz-Echevarría, Francisco Tejerina, Francisco José Parras, Pascual Balsalobre, Cristina Diez, Leire Pérez Latorre.

Hospital Universitari de Tarragona Joan XXIII (Tarragona): Francesc Vidal, Joaquín Peraire, Consuelo Viladés, Sergio Veloso, Montserrat Vargas, Miguel López-Dupla, Montserrat Olona, Anna Rull, Esther Rodríguez-Gallego, Verónica Alba.

Hospital Universitario y Politécnico de La Fe (Valencia): Marta Montero Alonso, José López Aldeguer, Marino Blanes Juliá, María Tasias Pitarch, Iván Castro Hernández, Eva Calabuig Muñoz, Sandra Cuéllar Tovar, Miguel Salavert Lletí, Juan Fernández Navarro, Jose Miguel Molina.

Hospital Universitario La Paz/IdiPAZ: Juan González-garcia, Francisco Arnalich, José Ramón Arribas, Jose Ignacio Bernardino de la Serna, Juan Miguel Castro, Luis Escosa, Pedro Herranz, Victor Hontañón, Silvia García-Bujalance, Milagros García López-Hortelano, Alicia González-Baeza, Maria Luz Martín-Carbonero, Mario Mayoral, Maria Jose Mellado, Rafael Esteban Micán, Rocio Montejano, María Luisa Montes, Victoria Moreno, Ignacio Pérez-Valero, Berta Rodés, Talia Sainz, Elena Sendagorta, Natalia Stella Alcáriz, Eulalia Valencia.

Hospital San Pedro Centro de Investigación Biomédica de La Rioja (CIBIR) (Logroño): José Ramón Blanco, José Antonio Oteo, Valvanera Ibarra, Luis Metola, Mercedes Sanz, Laura Pérez-Martínez.

Hospital Universitari MutuaTerrassa (Terrasa): David Dalmau, Angels Jaén, Montse Sanmartí, Mireia Cairó, Javier Martinez-Lacasa, Pablo Velli, Roser Font, Mariona Xercavins, Noemí Alonso.

Complejo Hospitalario de Navarra (Pamplona) María Rivero, Jesús Repáraz, María Gracia Ruiz de Alda, María Teresa de León Cano, Beatriz Pierola Ruiz de Galarreta.

Hospital Universitario de La Princesa (Madrid): Ignacio de los Santos, Jesús Sanz Sanz, Ana Salas Aparicio, Cristina Sarriá Cepeda, Lucio Garcia-Fraile Fraile, Enrique Martín Gayo.

Hospital Universitario Ramón y Cajal (Madrid): Santiago Moreno, José Luis Casado, Fernando Dronda, Ana Moreno, María Jesús Pérez Elías, Cristina Gómez Ayerbe, Carolina Gutiérrez, Nadia Madrid, Santos del Campo Terrón, Paloma Martí, Uxua Ansa, Sergio Serrano, María Jesús Vivancos.

Hospital General Universitario Reina Sofía (Murcia): Enrique Bernal, Alfredo Cano, Antonia Alcaraz García, Joaquín Bravo Urbieta, Ángeles Muñoz, Maria Jose Alcaraz, Maria del Carmen Villalba.

Hospital Universitario San Cecilio (Granada): Federico García, José Hernández, Alejandro Peña, Leopoldo Muñoz, Paz Casas, Marta Alvarez, Natalia Chueca, David Vinuesa, Clara Martinez-Montes, Fernando García, Carlos Guerrero-Beltran.

Centro Sanitario Sandoval (Madrid): Jorge Del Romero, Carmen Rodríguez, Teresa Puerta, Juan Carlos Carrió, Mar Vera, Juan Ballesteros, Oskar Ayerdi.

Hospital Clínico Universitario de Santiago (Santiago de Compostela): Antonio Antela, Elena Losada, Antonio Aguilera.

Hospital Universitario Son Espases (Palma de Mallorca): Melchor Riera, María Peñaranda, María Leyes, Ma Angels Ribas, Antoni A Campins, Carmen Vidal, Francisco Fanjul, Javier Murillas, Francisco Homar.

Hospital Universitario Virgen de la Victoria (Málaga): Jesús Santos, Crisitina Gómez Ayerbe, Isabel Viciana, Rosario Palacios, Carmen María González.

Hospital Universitario Virgen del Rocío (Sevilla): Pompeyo Viciana, Nuria Espinosa, Luis Fernando López-Cortés.

Hospital Universitario de Bellvitge (Hospitalet de Llobregat): Daniel Podzamczer, Elena Ferrer, Arkaitz Imaz, Juan Tiraboschi, Ana Silva, María Saumoy.

Hospital Costa del Sol (Marbella): Julián Olalla, Alfonso del Arco, Javier de la torre, José Luis Prada, José María García de Lomas Guerrero, Javier Pérez Stachowski.

Hospital Marina Baixa (VilaJollosa, Alicante): Concepción Amador.

Hospital General Universitario Santa Lucía (Cartagena): Onofre Juan Martínez, Francisco Jesús Vera, Lorena Martínez, Josefina García, Begoña Alcaraz, Amaya Jimeno.

Complejo Hospitalario Universitario a Coruña (Chuac) (A Coruña): Angeles Castro Iglesias, Berta Pernas Souto, Alvaro Mena de Cea.

Hospital Universitario Virgen de la Arrixaca (El Palmar): Carlos Galera, Helena Albendin, Aurora Pérez, Asunción Iborra, Antonio Moreno, Maria Angustias Merlos, Asunción Vidal.

Hospital Universitario Infanta Sofia (San Sebastian de los Reyes): Inés Suárez-García, Eduardo Malmierca, Patricia González-Ruano, Dolores Martín Rodrigo.

Complejo Hospitalario de Jaén (Jaén): Mohamed Omar Mohamed-Balghata, María Amparo Gómez Vidal. Nuestra Señora de Valme: Juan A Pineda, Juan Macías, Samuel Bernal. Unidad de Biología y Variabilidad del VIH.

Centro Nacional de Microbiología, ISCIII: Miguel Thomson, Elena Delgado, Sonia Benito, Vanessa Montero.

## Data Availability

The HIV-1 nucleotide sequences of our study population are available in GenBank with accession numbers MK588005–MK588395.

## Author Contributions

E-GK did the analysis and prepared the figures and the manuscript. AF and TK organized the data and built a database. NC, MA, PC, BA, and FG participated in the study design, performed the data collection across Spain, and provided critical comments about the manuscript. AH contributed to the study design and provided critical comments about the manuscript. DP performed the study design and supervised, and contributed to manuscript writing and editing.

## Conflict of Interest Statement

The authors declare that the research was conducted in the absence of any commercial or financial relationships that could be construed as a potential conflict of interest.

## Footnotes

1 https://www.hiv.lanl.gov/content/sequence/HIV/CRFs/CRFs.html

2 https://www.hiv.lanl.gov

3 http://tree.bio.ed.ac.uk/software/figtree/

4 https://www.hiv.lanl.gov/content/sequence/CONSENSUS/SimpCon.html

5 All clusters receive posterior probability support >0.82, expect for two clusters consisting of two sequences each, for which the posterior probability support was 0.76 and 0.79. The latter were included in the analysis since the confirmatory analysis was based on stringent sampling criteria.

6 http://www.mesquiteproject.org

## Abbreviations

CI: confidence interval
CRFs: circulating recombinant forms
CRF02_AG: circulating recombinant form 02_AG
HIV-1: human immunodeficiency virus type-1
MCMC: Markov chain Monte Carlo
MSM: men having sex with men
PLHIV: people living with HIV
t_MRCA_: time to the most recent common ancestor
